# Effects of Cosolvent on the Intermolecular Interactions between an Analyte and a Gold Nanostar Surface Studied Using SERS

**DOI:** 10.1021/acs.jpcc.4c04360

**Published:** 2024-10-02

**Authors:** Ryan D. Norton, Amanda J. Haes, Alexei V. Tivanski

**Affiliations:** † Department of Chemistry, University of Iowa, Iowa City, Iowa 52242, United States

## Abstract

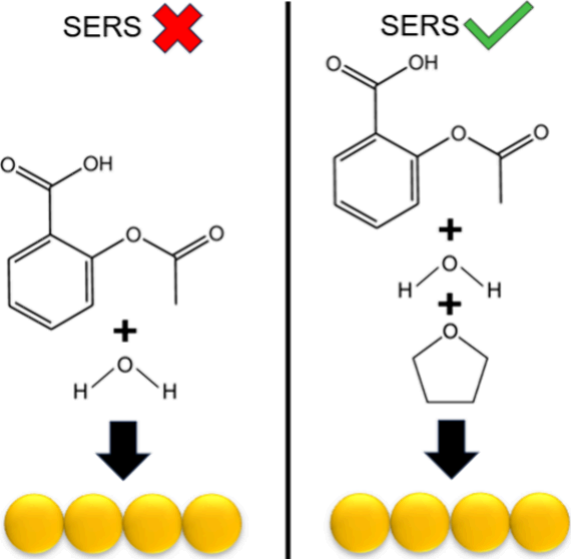

For surface-enhanced Raman scattering (SERS), reproducible solution-phase results are typically obtained using nanoparticles functionalized with surface-stabilizing molecules that can prevent the adsorption of analyte molecules with surface affinities lower than those of the stabilizing agent. Herein, we investigate the effects of intermolecular interactions between a nonthiolated analyte and cosolvent to facilitate and modulate analyte adsorption to gold nanostars. SERS, extinction spectroscopy, transmission electron microscopy (TEM), and density functional theory (DFT) calculations are employed in this regard. Tetrahydrofuran (THF) is utilized as a cosolvent in water to facilitate the detection of acetylsalicylic acid (aspirin) on anisotropic gold nanoparticles. Intermolecular interactions between the analyte, solvent, and surface are modulated by changing the solution composition to understand how THF facilitates the SERS detection of aspirin in THF–water cosolvents. SERS signals for 5 mM aspirin arise only in the presence of THF at and above 60 mM, while no signal with or without THF below 60 mM is observed. SERS detection of aspirin is hypothesized to depend on THF forming a hydrogen-bonded complex with aspirin that reduces aspirin hydrophobicity, thus stabilizing the acid form of the molecule and allowing it to weakly interact with the gold nanoparticles. The aspirin–THF complex adsorbs to the gold surface through π-orbital overlap between the aromatic ring and gold, where additional THF weakens orbital overlap. Understanding the mechanism by which organic cosolvents facilitate the SERS detection of nonthiolated analytes such as aspirin, in aqueous media, allows other cosolvents, nonthiolated analytes, and other surfaces to obtain a SERS signal in a variety of systems.

## Introduction

Surface-enhanced Raman scattering (SERS) often utilizes anisotropic metal nanoparticles (e.g., nanostars) to generate strong electromagnetic fields near interfaces for surface-selective signal enhancement.^1−8^ The electric field gradient and strength depend on nanoparticle morphology and govern the enhancement for molecular detection.^1,2,7,9^ Because monodisperse solution-phase nanoparticles contain surface-stabilizing agents to decrease/prevent cluster formation,^10^ SERS enhancements are lessened when using these SERS substrates because the field drops off rapidly from the metal interface and the stabilizing agent limits or even completely prevents molecular adsorption or binding.^11^

Approaches that facilitate the molecules’ experience of the strong electric fields required for SERS are common. This is especially important for promoting the adsorption of nonthiolated molecules and facilitate SERS signals. Various mechanisms include exploitation of nanoparticle morphology,^2,6^ nanoparticle composition,^12,13^ nanoparticles separation distance,^14−16^ surface-stabilizing agent identity,^4^ and medium composition.^17−19^ For instance, gold nanostars, which exhibit small radius of curvature features that give rise to large electric fields and thus large SERS signals, can be synthesized and stabilized by Good’s buffers.^4^ Surface activation has been previously shown to open a binding pocket, a small space on the surface not protected by a stabilizing agent, for molecules with weak surface affinity to reach and directly interact with the surface by selectively protonating a nitrogen in the piperazine ring in the Good’s buffer species.^4,5^

Previously, cosolvents were used to understand how SERS intensity is affected by analyte solubility in cosolvent(s),^17^ analyte intermolecular interactions with a solvent and surface,^18,19^ and coadsorption of an analyte monolayer and solvent to a surface.^20^ Cosolvents were shown to modulate analyte affinity to the surface by directly binding to the molecule to form complexes,^20^ change the physical properties of the analyte such as the hydrophobicity of a complex,^21^ or facilitate transport through a solvent layer formed near the nanoparticle surface.^22^ Solvent composition also influences the preferred intermolecular interactions for solvation such as the use of polar vs nonpolar solvents to solvate an organic acid or ether.^23,24^ Molecular concentration also affects solvation, as a lower water to analyte molar ratio was shown to increase analyte–analyte interactions such as organic acid dimers.^25,26^

Overall, the effects of cosolvents for SERS and solution-phase nanoparticles are not fully understood.^17,19^ Fundamental knowledge regarding mechanisms is limited by how cosolvents bind to or solvate the analyte, surface, and surface-stabilizing agents, where cosolvents are known to affect analyte detection and orientation on a surface.^17−19^ Addressing these knowledge gaps has the potential to yield better experimental designs that improve the predictability of how the solvent affects an analyte and SERS signal.

In this study, aspirin, an analyte with weak affinity to a gold surface (i.e., weaker than the surface stabilizing agent–gold interaction), is selected because it contains carboxylate, aromatic ring, and ester groups that exhibit descending yet collective affinity to gold. The presence of multiple functional groups capable of interacting with the surface gives the flexibility to study how a cosolvent can affect the expected surface adsorption. Next, tetrahydrofuran (THF) is used as a cosolvent with water because (1) THF is 100% miscible in water, (2) aspirin solubility is high in THF (∼2.8 M due to hydrogen bond formation between THF ester and aspirin carboxylic acid) versus in water (∼18 mM),^27,28^ (3) THF has no significant affinity to gold, and (4) the characteristic vibrational modes of THF do not overlap with aspirin. In doing so, the SERS signal for 5 mM aspirin was only observed when THF is present at or above 60 mM, a result attributed to the likely modulation of intermolecular interactions in solution, yielding formation of hydrogen-bonded aspirin–THF complexes that facilitate transport and adsorption of aspirin–THF to the gold surface nearly parallel to the surface through π-orbital overlap with the aromatic ring.^17^ The use of THF–water solvents extends SERS detection to molecules with low water solubility and weak surface affinity through under-explored solvation tuning.^17^ Further increasing THF concentration, beyond the threshold required for SERS, results in weakening the π-orbital overlap between aspirin and gold, thus weakening aspirin surface adsorption and resulting in a greater understanding for how cosolvent affects molecular adsorption beyond facilitation. These results are expected to serve as a foundation for exploring the impacts of other cosolvents and the detection of molecules with weak surface affinity (i.e., nonthiolated molecules) by SERS.

## Experimental Methods

### Gold Nanostar Synthesis

All glassware was cleaned in aqua regia (3:1 hydrochloric acid/nitric acid) and rinsed with ultrapure water (18.2 MΩ·cm^–1^ obtained from a Barnstead Nanopure System) several times before drying with filtered air. Gold nanostars stabilized by 3-[4-(2-hydroxyethyl)piperazin-1-yl]propane-1-sulfonic acid (EPPS) were synthesized according to prior published procedures.^2^ Briefly, the pH of 100 mL of 100.0 mM EPPS was increased to 7.45 (±0.01) with 1.0 M sodium hydroxide. Next, 80 mL of 100 mM EPPS was filtered using a 45 μm PTFE syringe filter and then stirred for 10 min to reach a homogeneous mixture followed by the addition of 800 μL of 20 mM gold(III) chloride trihydrate. The resulting mixture was stirred for 10.0 min. Gold nanostar growth occurred in an undisturbed solution at room temperature (20–23 °C). After 12+ hours, the resulting gold nanostars solution was centrifuged at 1800 × *g* for 45 min at room temperature, and then repeated using the supernatant. The concentrated gold nanostars were combined, redispersed in 20 mM EPPS by vortexing for 30 s, and centrifuged twice at 1800 × *g* for 45 min. The supernatant was removed before the soft pellet was redispersed in 5 mM EPPS by vortexing for 20 s, twice. The gold nanostar solution at typical concentration of 1.0–1.6 nM was stored at 4.0 °C until use. Gold nanostar concentration was determined using Beer’s law with a previously determined extinction coefficient as described in literature (ε = 2.3 × 10^9^ M^–1^·cm^–1^).^29^

### Raman Sample Preparation and Measurements

Raman spectra were collected using a semihome-built Raman microscope (Olympus BX51) coupled with an ExamineR 785 (Intevac) Raman detector. Raman spectra were collected at 785 nm with a laser power of 32.7 mW, 15 s integration time, and 10 averages. All percentages of THF (99.9% purity, Fisher Scientific) are reported in terms of weight unless otherwise stated. All water used throughout this study is ultrapure water. The mass percentage of THF in THF/water solutions was varied from 5% to 99.9% THF. Each solution, which includes a varying ratio of two cosolvents (i.e., THF and water), was vortexed for 10 s prior to Raman spectral analysis. All samples labeled by % THF or M THF are prepared with only water and THF. Collectively, 45 samples were prepared for 15 concentrations measured in triplicate for THF in water.

Solutions of 5 mM to 1.5 M acetylsalicylic acid (aspirin, C_9_H_8_O_4_) in neat THF were prepared by mass and vortexed for up to 30 s until all aspirin was fully dissolved. Each sample was vortexed for 10 s prior to collecting Raman spectra. Raman intensity calibration curves for aspirin were generated for aspirin in neat THF as the solubility limit of aspirin in water is limited to ∼18 mM.^28^ Collectively, 30 samples were prepared with aspirin in THF with 10 concentrations measured in triplicate.

Solutions of 0–2 M aspirin were serially prepared in 30% THF in water. To prepare these samples, aspirin was weighed and added to solutions of 30% THF with each sample made in triplicate, vortexed for 60 s, and equilibrated for 12+ hours. Upon vortexing, a second, immiscible liquid phase forms in all solutions containing at least 0.15 M aspirin due to the solubility limit of the mixed solution. When a new liquid phase was generated, the less dense phase was removed and analyzed separately from the new denser phase. Each phase was vortexed for 10 s prior to Raman spectral collection. Any sample that generated bubbles lasting longer than 30 s after this vortex was prepared again as this indicated the poor isolation of the two liquid phases. Collectively, 45 samples were prepared with aspirin in 30% THF/water solution, with 15 concentrations measured in triplicate.

### SERS Sample Preparation and Measurements

Before use, 1.37 nM gold nanostars^29^ in 5 mM EPPS was centrifuged twice at 1800 × *g* for 45 min. The supernatant was then removed and replaced with ultrapure water to slowly decrease the EPPS concentration in solution. After supernatant removal the second time, the nanostars were redispersed in water such that the nanostar concentration was 13.7 nM, as determined via extinction spectroscopy and Beer’s law with the previously determined extinction coefficient.^29^ A previously established approach was utilized to activate the surface-stabilizing agent on the gold nanostars,^4^ where 634.5 μL of pH 3.8 nitric acid was added to 65.5 μL of the 13.7 nM nanostars to a final gold nanostar concentration of 1.3 nM at pH of 3.8. This sample was vortexed for 10 s, and then extinction spectra were collected to monitor the wavelength at the maximum extinction (λ_max_). The λ_max_ associated with the hybridized nanostar branch resonance at 760 nm blue-shifted with increasing time.^4^ Once the λ_max_ blue-shifted by 5 nm, to minimize nanostar morphological changes and resulting effects on SERS signal,^4^ the sample was quenched with 100 μL of pH 10.5 sodium hydroxide. The quenched solution was immediately vortexed for 10 s. We note that the solution pH is not expected to affect analyte protonation state in aqueous media.^30^

Next, activated gold nanostars were dispersed in 0–2 M THF in water by adding 80 μL aliquots of either 85% THF or both 70% THF and the new denser separated phase described above. Each sample was again vortexed for 10 s before extinction, and SERS spectra were simultaneously collected. THF percentage is different between sample and reference (i.e., identical samples except that aspirin was not included) to control the volume of solution such that nanostars concentration remains constant throughout the study. Volumes of the standard solution, 70% THF, and the dense, separated phase are controlled in all samples, so aspirin concentration remains constant. Samples not containing aspirin, reference samples, were prepared by varying THF concentration using an 85% THF standard solution. The magnitude of the hybridized branch plasmon was measured at the wavelength corresponding to the maximum signal relative to the average signal between 550 and 575 nm.

SERS samples were prepared using the new denser phase along with a 70% THF in water solution to vary solvent composition while retaining a constant aspirin concentration of 68 mM and a constant 0.93 nM activated nanostar concentration. The dense phase volume added was held constant at 50 μL, and a 70% THF–water stock solution was added in varying volumes to 800 μL of activated gold nanostars. Water was added to control the final solution volume and the final concentrations of aspirin, nanostars, and THF. The resulting THF concentrations ranged from 0–0.043 mole fraction THF. The sample was vortexed for 10 s, then localized surface plasmon resonance (LSPR) and SERS spectra were collected simultaneously every 35 s for 45 min to determine equilibration time for the SERS signal associated with aspirin to reach its maximum. The maximized and stabilized intensity was reached within 12 min, so all spectra analyzed in this experiment represent data collected after this time with each sample vortexed for 10 s again before data collection. After 30 min, the gold nanostars began to aggregate so spectra collected after this time were not used.

### Simultaneous Extinction Spectroscopy and SERS Measurements

SERS spectra were collected using the same system previously reported for Raman, along with an Integrated Photonic Solutions fiber optically coupled laser operated at an excitation wavelength of 785 nm, a laser power of 35.2 mW, and integration times of 35 s. Higher integration times were utilized for SERS to account for the significantly lower analyte concentrations relative to those from Raman. Ten spectra were averaged for both methods. Raman intensities were collected in terms of photon counts (cts) but are reported in units of cts·mW^–1^·s^–1^ to account for differences in laser power and integration times. Spectral analysis was performed using the first and second derivatives. Briefly, vibrational frequencies were determined by the zero-crossing points (ZCPs) of the first derivative of the spectra for major vibrational modes.^4^

First derivatives with no smoothing were obtained from raw data for intense features (signal-to-noise ratio greater than 5). First derivatives using Savitsky-Golay smoothing with an 11-point window and second-order polynomial were obtained for features with low intensity (signal-to-noise ratio less than 5), where noise can affect the accuracy of determining the position of the vibrational center.^4^ These low intensity modes had either multiple ZCPs for the same feature (the maximum of the feature is affected by noise) or no ZCPs (nearly crosses zero) and required smoothing to resolve the true vibrational frequency. The true vibrational frequency was cross-referenced against a Gaussian fit of the observed feature. Second derivatives were utilized to determine the vibration frequency associated with weak, or overlapping, features within larger, asymmetric features and relative intensities. An 11-point window, second-order polynomial, and Savitsky-Golay smoothing were used to generate second-derivative spectra. The magnitude of the inverse second derivative, from a high-energy maximum to a low-energy minimum, can be used to represent the intensity of a feature in the raw data and to resolve overlapping features. Magnitude changes were analyzed for spectral trends as a function of solvent composition, and variations in magnitude for each significant vibrational mode were used to assess impacts of intermolecular interactions. Features in the Raman spectra with a full-width at half-maximum (FWHM) below 8 cm^–1^ (or below 10 cm^–1^ for SERS), intensity below three times the noise level (calculated as the standard deviation, between 1900–1800 cm^–1^, where no vibrational modes are present), or not identifiable as THF or aspirin, were considered artifacts and/or not reproducible. Artifacts were excluded from the report but may be included during spectral deconvolution.

Extinction spectra were collected from 0.87 nM gold nanostars, in either water or varying concentrations of THF in water, using a B&W Tek UV–vis spectrometer with a Mikropack halogen light source. Extinction spectra were collected to determine nanostars stability and morphology through analyzing the plasmonic structure.^31^ LSPR is monitored by extinction spectroscopy through the change in λ_max_, FWHM, and extinction magnitude at λ_max_. Data were collected with a path length of 0.8 cm across a wavelength range of 380–1100 nm with an integration time and number of averages at 35 s and 10, respectively. Spectra were analyzed by quantifying corresponding FWHM for all vibrational modes, first and second derivatives. The FWHM is utilized along with corresponding intensity and vibrational frequency to identify whether an observed feature is a real vibrational mode or nonreproducible signal from a variety of sources, including cosmic rays or contamination.^32,33^ The first derivatives are utilized to identify the vibrational frequency of each observed feature. The second derivative separated the plasmonic contributions of the hybridized, branch plasmon and any plasmon arising from nanostars aggregation.^4^ The second derivative was used to determine the frequency of SERS vibrational modes whenever the first derivative did not yield a ZCP for either the hybridized branch or the electromagnetic coupling plasmons.

### Transmission Electron Microscopy (TEM) Measurements

Nanostar morphology and dimension were determined using TEM and as a function of solvent and analyte concentration and/or composition. Data were collected with a Hitachi HT7800 TEM equipped with AMT Nanosprint15 high-resolution camera at 80 kV acceleration voltage and 100,000× magnification. Nanoparticles were synthesized and prepared following the previously described protocol before being diluted by 50% using ethanol. The solution was vortexed for 10 s before depositing 200 μL dropwise onto 400 mesh copper grids coated with Formvar and carbon (Ted Pella) and then dried at room temperature. A new drop was deposited only after the previous drop fully dried. TEM images were processed and analyzed using ImageJ to discern the branch length, Feret diameter, and radius of curvature at the branch tip following the approach described previously.^2,34^ At least 100 individual gold nanostars were analyzed per prepared TEM grid, with multiple measurements collected per each star. A minimum of 200 measurements were collected for both branch length and radius of curvature before comparing the average and standard deviations between stars exposed to varying compositions of water, THF, and aspirin.

### Density Functional Theory (DFT) Calculations

Electrostatic potential energies and equilibrium geometries were calculated for systems of aspirin in THF/water to determine how the solvent composition affected the electron distribution. Aspirin, THF, and water molecules were explicitly added. Four gold atoms were explicitly added with spatial coordinates fixed, emulating a (111) crystal face found in gold nanostar branches to simulate a gold interface. Spartan’20 (Version 1.1.4) was used to minimize system energy based on equilibrium geometry. First, the Merck molecular force field was used followed by DFT with the B3LYP/6-31G* level of theory.^35,36^ This level of theory was chosen to optimize aspirin and THF molecular structure and electrostatic potential near a surface.^4^ Sequential calculations ensured that the energy minimization function converged, leading to equilibrium geometries. Each system was optimized from multiple different initial settings including varying the atom/molecule position and orientation before starting the calculations.^37^ All systems reported were calculated with water as an implicit solvent using the SM8 quantum mechanical aqueous continuum solvation model.^38^

## Results and Discussion

### Exploiting Solute–Solvent Interactions to Improve SERS Detection of Aspirin

**Figure 1:**
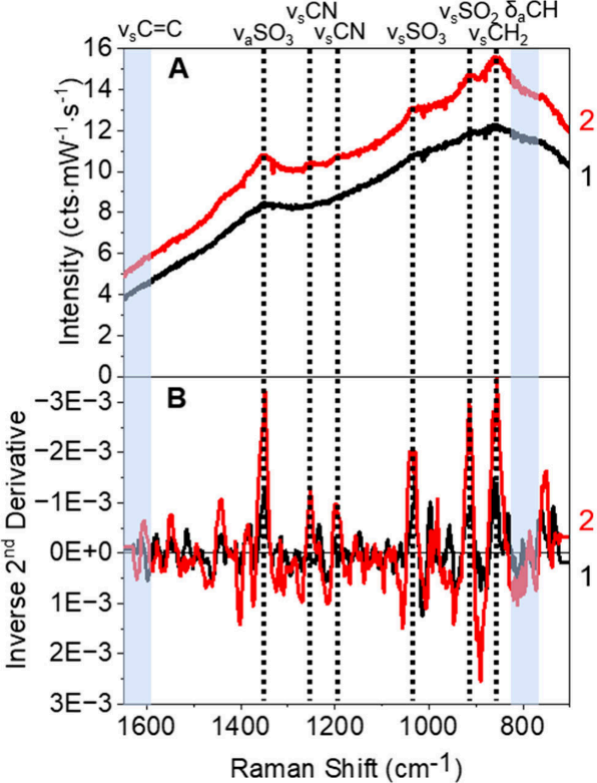
(A) SERS spectra and (B) corresponding inverse 2^nd^ derivatives spectra of 0.37 nM activated gold nanostars in water without aspirin (1, black lines) and 0.37 nM activated gold nanostars in water with 5 mM aspirin (2, red lines). The blue stripes highlight key spectral regions where aspirin modes are expected to be located. Vertical black dotted lines at 1352, 1253, 1197, 1037, 915, and 856 cm^–1^ correspond to the ν_a_SO_3_, ν_s_CN, ν_s_CN (2^nd^ nitrogen in the piperazine ring), ν_s_SO_3_, ν_s_SO_2_, and ν_s_CH_2_ assigned to the EPPS on activated, gold nanostar surfaces.

Figure 1A shows SERS spectra for surface-activated gold nanostars with 5 mM aspirin and a control of nonactivated nanostars with no aspirin. To further distinguish these spectra from background and noise features, corresponding second derivative spectra are shown in Figure 1B. Several significant vibrational modes can be identified at 1350, 1252, 1198, 1035, and 914 cm^–1^ in the second derivative spectra and can be assigned to ν_a_SO_3_, ν_s_CN, ν_s_CN (2^nd^ nitrogen in the piperazine ring), ν_s_SO_3_, and ν_s_SO_2_, respectively.^4^ The presence of SO_3_ and CN vibrational modes correlates to the activated surface chemistry, EPPS, and confirms that the gold nanostars were successfully activated.^4^ This is confirmed from the appearance of EPPS vibrational modes that, similar to *N*-2-hydroxyethylpiperazine-*N*′-2-ethanesulfonic acid, are not present before activation.^4^ Because the SERS spectrum of 5 mM aspirin does not display any vibrational modes for aspirin around 1609 cm^–1^ (ν_C=C_) and between 750 and 850 cm^–1^ (δ_CH_), shown by blue boxes in Figure 1, aspirin is not detected even though the gold nanostars are activated.^4,17,39−42^ The lack of analyte specific features suggests that aspirin has not adsorbed to gold and/or the vibrational modes are not oriented in a matter to satisfy surface selection rules. It should be noted that 5 mM aspirin should be detected using SERS.^17,39,40^ In particular, our group previously used similar acid activation of gold nanostars to detect 5 mM benzene, which exhibits only London dispersion-like interactions with gold.^4^ Thus, two possible mechanisms that prevent aspirin from experiencing large electric fields near the gold surface are considered. These include steric or charge restrictions based on the size of the binding pocket on the gold surface formed when EPPS is activated and/or the presence of a structured water layer around the nanoparticle surface that inhibits the transport and adsorption of hydrophobic aspirin to gold.^4,5,43,44^

**Figure 2:**
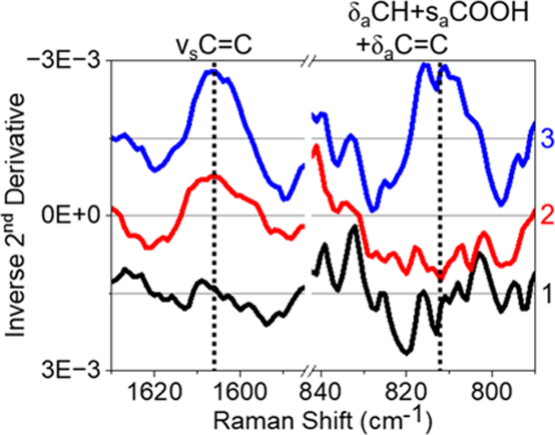
Inverse 2^nd^ derivative SERS spectra for of 0.93 nM activated gold nanostars in water (1, black line), with 5 mM aspirin and 10 mM THF in water (2, red line), and with 5 mM aspirin and 60 mM THF in water (3, blue line). Spectra 1 and 3 were offset relative to spectrum 2 for clarity (+0.0015 for 1 and −0.0015 for 3). Vertical black dotted lines correspond to aspirin’s unique vibrational modes, with assignments above each line.

Previously, the possibility of densely packed solvent layer formation, such as proposed here, was hypothesized for gold nanospheres,^43,45^ zwitterion-stabilized silica nanospheres,^22^ and ionic transport through a solvent layer.^22^ We assert that a similar effect prevents the adsorption of aspirin into gold nanostars. Several pieces of indirect evidence support the presence of the water layer, which prohibits aspirin adsorption. To elucidate this mechanism, analyte solubility is varied by adding THF with a fixed aspirin concentration to maximize SERS intensities.^17^Figure 2 shows representative second derivative spectra in the range where aspirin signal should be observed for activated stars without aspirin as well as with 5 mM aspirin in 10 mM and 60 mM THF. No aspirin signal is detected when THF concentration is less than 60 mM; however, two aspirin modes appear at 1606 and 811 cm^–1^, representing the ν_s_C=C and a combination band δ_a_CH + s_a_COOH + δ_a_C=C, respectively, when the THF concentration is 60 mM or greater.^39,46^ The presence of the vibrational mode at 811 cm^–1^ for aspirin suggests that THF facilitates the direct adsorption of aspirin to the gold surface.^39,46,47^ A control SERS measurements of 5 mM aspirin in water alone (see Figure 1) and in 10 mM THF (see Figure 2) rule out the possibility of steric restriction based on the binding pocket size previously considered, as no aspirin signal was detected in either measurement. SERS measurements of gold nanostars in varying THF concentration without aspirin support that THF presence does not significantly affect EPPS on the surface. Thus, the use of THF–water cosolvents is hypothesized to facilitate the detection of aspirin using SERS by forming an aspirin–THF complex via hydrogen bond between the carboxylic acid in aspirin and ether group in THF. The ratio of aspirin to THF, observed when comparing the spectra of 5 mM aspirin in 10 and 60 mM THF (i.e., 1:2 and 1:12, respectively), likely indicates that the extended solvation layer of aspirin (interactions beyond forming the hydrogen bonded complex) further increases the complex’s hydrophilicity, which increases its residence time near the metal surface to enable the SERS detection of aspirin. The complex and resulting solvation layer (amount of THF present likely dependent on aspirin/THF ratio) is hypothesized to increase aspirin hydrophilicity and in turn facilitate the movement of aspirin through the potential organized water layer near the particle surface, thus reaching and adsorbing to the surface to enable the SERS detection of aspirin. This can be supported by THF being 100% miscible in water, hydrophilic, and the relative solubility limits of aspirin in water and THF implying that aspirin is primarily solvated by THF instead of water. Stronger intermolecular interactions between THF and aspirin, relative to aspirin and water, lead to aspirin being preferentially solvated by THF thus generating a hydrophilic solvation layer that facilitates the transport of aspirin through an organized solvent layer to approach the zwitterionic surface-stabilizing agents and the nanoparticle surface enabling surface adsorption.

The formation of aspirin–THF complexes can be supported by the vibrational mode identification in the SERS spectra. Only two modes are detectable for 5 mM aspirin in 60 mM THF (Figure 2). While these modes support aspirin detection by SERS, the adsorption mechanism can be confirmed only by increasing the aspirin concentration in solution 10-fold. This 10-fold increase is shown to not influence the gold nanostar morphology. This is important because the concentrations of THF and aspirin were increased to over 600 mM and 50 mM (10x the concentrations in Figure 1), respectively. TEM analysis reveals that the morphology, branch length, and radius of curvature of gold nanostars do not change with the addition of 0–0.043 mole fraction THF or 5–68 mM aspirin. Importantly, the presence of THF is confirmed by small changes in the extinction maximum wavelength from the gold nanostars, which is consistent with bulk changes in refractive index (Figure S1). LSPR λ_max_ and fwhm are the primary parameters used to infer the morphology of the nanostars from extinction spectroscopy.^2^ Here, adding 0.043 mole fraction THF results in the 14 nm total red-shift in the low energy plasmon relative to our nanostars in water, which is attributed to the bulk refractive index increasing linearly with THF concentration and a smaller 5 nm red-shift upon aspirin–THF adsorption. It is important to note that this spectral change does not arise from morphological changes obtained via TEM (Figure S2). Because the concentration of aspirin on gold is significantly higher than the solution concentration,^48^ up to 6 M THF and 2 M aspirin in water are used to understand the key intermolecular interactions that mimic the experimental conditions where aspirin is SERS-active.^48^

### Understanding the Role of Solute–Solvent Intermolecular Interactions between Aspirin, THF, and Water

To further understand solution compositional effects on intermolecular interactions between THF, aspirin, and the surface, high concentration (i.e., several molar) solutions of aspirin and THF in water are evaluated. Aspirin concentration is increased in THF–water cosolvent, which induced liquid–liquid phase separation as the solubility limit of the mixed solution is exceeded (Experimental Methods). Both phases are analyzed using normal Raman scattering to understand the intermolecular interactions present in solution and to quantify the amount of the species in each phase. These calibration curves are generated in two-component systems of varying THF concentrations in water (Figure S3) and varying aspirin concentrations in THF (Figure S4) before plotting the Raman intensity as a function of aspirin or THF concentration.

The two phases are analyzed to understand the intermolecular interactions present in high concentration solutions. THF complexation with and solvation of aspirin decreases the hydrophobicity of aspirin in solution.^21^ The three-component system of aspirin, THF, and water generates a new, denser, liquid phase as the hydrophobicity of aspirin changes based on the molecular orientation and composition of the solvation layer.^21^ Raman spectra identify key aspirin modes, ν_CC_ at 1609 cm^–1^, δ_CH_ + δ_COOH_ at 785 cm^–1^, and δ_CH_ at 754 cm^–1^ in both phases with key THF modes, ν_CC_, ν_CO_, and ν_COC_ in the feature around 914 cm^–1^, and s_CH_2__ modes in the feature around 1450 cm^–1^ in both phases as well (Figure S5).^46,49^ The absence of the carboxylate features at 1535 cm^–1^ in Raman spectra support that aspirin is protonated in both phases.^50^ The observed vibrational shifts for vibrational modes containing oxygen in aspirin and THF (∼3 cm^–1^) are smaller than the 7–14 cm^–1^ shift previously observed,^46,49,51^ therefore further supporting that THF forms a hydrogen bonded complex with aspirin. The relative signal intensities for aspirin and THF indicate that the new phase contains more THF and aspirin than originally added and thus more closely resembles the system observed at a surface than in solution. Calibration curves suggest that the concentrations of aspirin and THF are 65 mM and 1.9 M (0.0392 mole fraction THF), respectively, for the original phase and 1.5 M aspirin and 5.8 M THF (0.165 mole fraction THF) in the new denser phase. These concentrations correspond to molar ratios of 1 aspirin/30 THF/722 water for the original phase and 1 aspirin/4 THF/20 water for the new denser phase.

The molar ratio for aspirin/THF/water, in the dense phase, provides the basis for DFT calculations for 1 aspirin, 4 THF, and 20 water molecules to understand how THF, interacts with aspirin at high concentrations.^21^ DFT calculations suggest the presence of a strong hydrogen bond between THF and aspirin, while the other THF molecules orient around aspirin with part of the THF oxygen exposed to the bulk solution (Figure S6). This strong hydrogen bond stabilizes the carboxylic acid thereby generating a complex between aspirin and THF while the other THF solvating aspirin are more easily displaced based on the relative interaction energies. The orientation and number of THF molecules surrounding the aromatic ring of aspirin can decrease the hydrophobicity of solvated complex relative to deprotonated aspirin in water.^21^ The DFT calculations and Raman results described above thus support the hypothesis that THF forms a complex with aspirin in solution, thus reducing the hydrophobicity of aspirin and enabling adsorption.

### Determining the Effects of Cosolvent and Solute–Solvent Intermolecular Interactions on Molecular Adsorption through SERS

**Figure 3:**
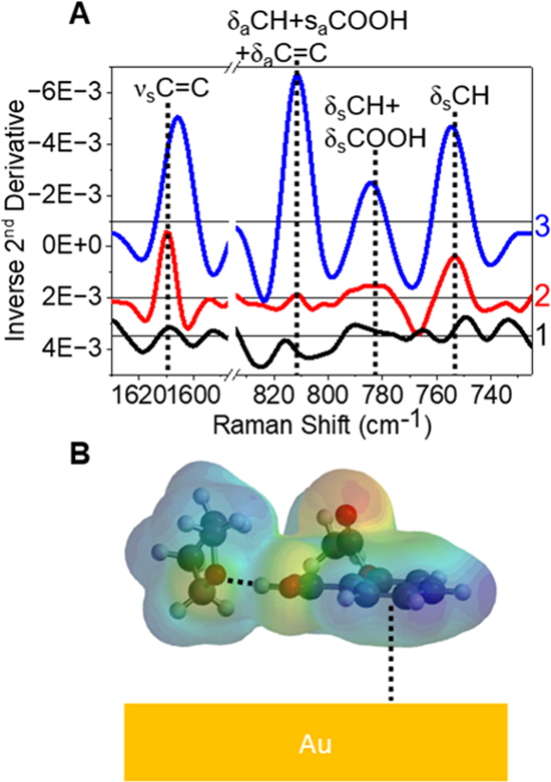
(A) Inverse 2^nd^ derivative spectra for SERS of 0.93 nM gold nanostars with 1 M THF in water (1, black line), Raman of 68 mM aspirin with 1 M THF in water (2, red line), and SERS of 0.93 nM gold nanostars with 68 mM aspirin and 1 M THF in water (3, blue line). Vertical dotted black lines correspond to aspirin’s unique vibrational modes, with assignments above each line. (B) Possible structure of aspirin binding on a gold surface through the aromatic ring (illustrated via black dotted line), where THF coordinates to aspirin as modeled through DFT calculations. Inverse 2^nd^ derivative spectra for SERS were offset for clarity (+0.0035 for 1, +0.002 for 2, and −0.001 for 3). Water molecules are not shown for clarity.

The aspirin–THF complex surface adsorption mechanism depends on the THF concentration, as revealed by SERS, extinction spectroscopy, and DFT calculations. While the aspirin–THF complex could interact with the gold surface through carboxylate, aromatic ring, and ester groups (listed in descending affinity to gold), surface stabilization from aromatic ring–gold interactions is indicated. As shown in Figure 3, high concentrations of aspirin and THF are used to determine vibrational mode assignments and adsorption mechanisms as well as to separate SERS from normal Raman features. Figure 3A shows representative Raman and SERS of 68 mM aspirin in 1 M THF, respectively, compared to SERS of nanostars in 1 M THF without aspirin to understand the mechanism of the aspirin–THF complex adsorption on gold. Several observations support that an aspirin–THF complex is observed near the gold surface. First, the presence of a vibrational mode at 810 cm^–1^ in SERS spectra supports the premise that aspirin is adsorbed to gold as this feature, which is a combination mode arising from δ_a_CH + s_a_COOH + δ_a_C=C, is not observed in normal Raman. Second, SERS spectra also show modes at 1605, 785, and 752 cm^–1^ that can be attributed to aspirin (ν_s_C=C at 1605 cm^–1^, δ_s_CH + δ_s_COOH at 785 cm^–1^, and δ_s_CH at 752 cm^–1^, respectively). The observation of vibrational modes that arise from COOH and not carboxylate further supports the notion that THF, not water, preferentially stabilizes aspirin. Third, the absence of the most energetically favored carboxylate–gold interaction further suggests that aspirin likely adsorbs to gold via the aromatic ring.

The adsorption mechanism between the aromatic ring of aspirin and the gold surface is further supported by LSPR spectroscopy. First, an additional 5 nm red-shift in the lower energy plasmon mode is observed when 68 mM aspirin in 0.039 mole fraction THF is added (Figure S7). This response is similar to the 3–5 nm red shift previously observed for benzene derivatives upon adsorption.^4,5^ Because the gold nanostars do not undergo significant structural changes under these conditions (see SI for more information) and do not undergo electromagnetic coupling, this additional wavelength shift is attributed to a ∼0.013 increase in local refractive index when aspirin–THF complexes adsorb to surface activated EPPS-stabilized gold nanostars.^52^ This slight increase in the local refractive index is reasonable given the high surface density of the surface stabilizing agent EPPS on gold and the similar refractive indices of organic species.

Equilibrium geometries, as determined from DFT, further support that aspirin is adsorbed nearly parallel to gold through the aromatic ring. Figure 3B reveals that the aspirin–THF hydrogen-bonded complex adsorbs nearly parallel to the gold surface through the aromatic ring via π-orbital overlap with gold. This orientation is consistent with the intense SERS spectral features associated with this orientation geometry at 1605, 810, 785, and 752 cm^–1^ (Figure 3A). Furthermore, the ν_s_C=C red-shifts from 1609 to 1605 cm^–1^ (normal Raman to SERS), indicating that the aromaticity of the ring is decreasing, likely through surface interactions. In addition, the relative ratios of SERS to normal Raman intensities allow for identification of vibrational modes that are selectively enhanced via surface selection rules.^8^ In Figure 3A, the ν_s_C=C and out-of-plane aromatic ring CH deformations are selectively enhanced, further supporting that the aromatic ring in aspirin is parallel to gold and that the C–H groups are near the metal surface.

**Figure 4:**
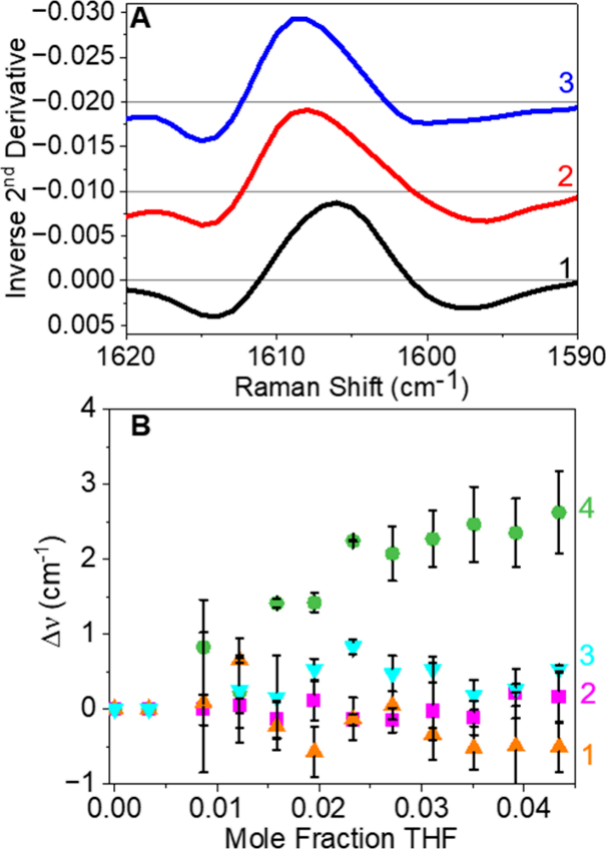
(A) Inverse 2^nd^ derivative spectra for SERS of 68 mM aspirin in 0.009 (1, black line), 0.023 (2, red line), and 0.043 (3, blue line) mole fractions of THF in water. (B) SERS Raman shifts relative to 0 M THF as a function of THF in water mole fraction for several selected aspirin modes: δ_s_CH + δ_s_COOH (784 cm^–1^, 1, orange triangles), δ_a_CH + s_a_COOH + δ_a_C=C (811 cm^–1^, 2, pink squares), δ_s_CH (754 cm^–1^, 3, magenta triangles), and ν_s_C=C (1609 cm^–1^, 4, green circles). Error bars represent one standard deviation from three repeated measurements. Inverse 2^nd^ derivative spectra for SERS were offset relative to spectrum 1 for clarity (−0.01 for 2 and −0.02 for 3).

**Figure 5:**
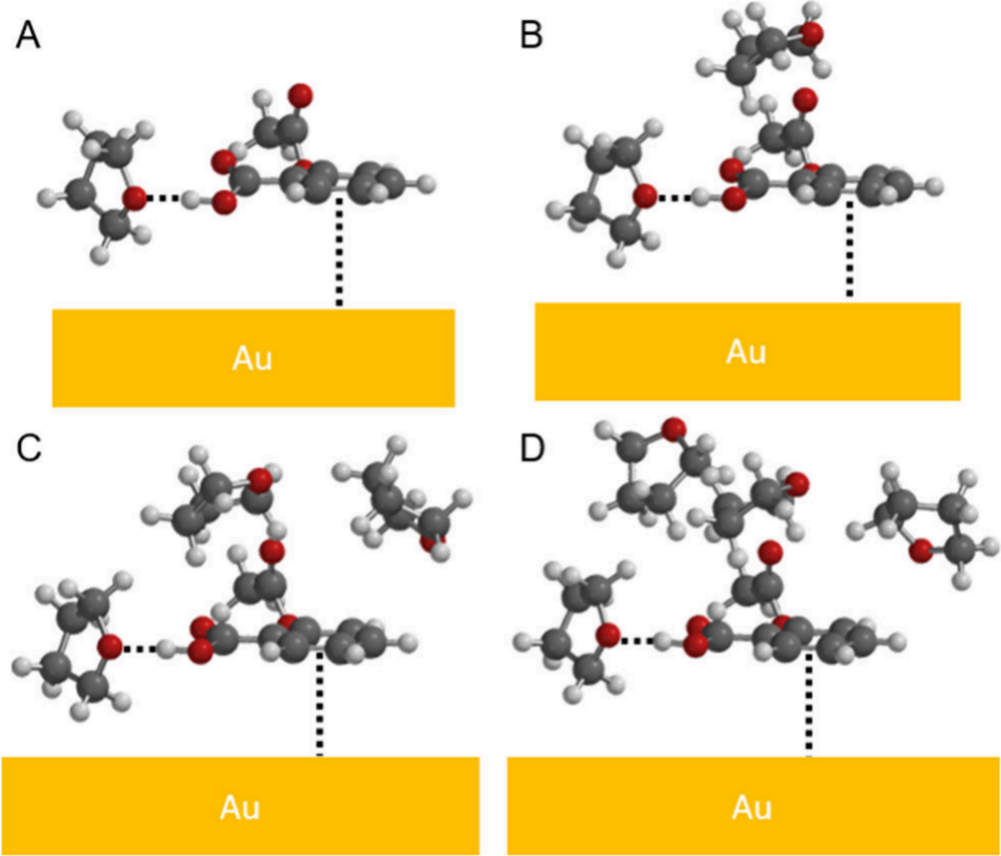
DFT calculations depicting equilibrium geometries for how (A) 1 THF, (B) 2 THF, (C) 3 THF, and (D) 4 THF molecules interact with a single aspirin near a gold surface in water. Water molecules are not shown for clarity.

Now that the intermolecular driving forces between the aspirin–THF complex and the gold surface are understood, the role of THF is evaluated. It is likely that THF preferentially solvates aspirin over the gold surface and that these interactions collectively weaken the π-orbital overlap between aspirin and gold. This is supported through the analysis of SERS spectral features and DFT results. Figure 4A shows how the ν_s_C=C spectral feature for aspirin changes when the mole fraction for THF is increased from 0.009 to 0.043. While changes in the vibrational frequency are observed, the intensity remains constant. As shown in Figure 4B, the ν_s_C=C vibrational frequency systematically blue-shifts to a total of 2 cm^–1^ as THF concentration increases, while that for δ_a_CH + s_a_COOH + δ_a_C=C, δ_s_CH + δ_s_COOH, and δ_s_CH remain relatively constant. This small yet systematic blue-shift in the ν_s_C=C suggests that the electron density in the aromatic ring for aspirin is increasing with increasing THF and that the solvation environment and orientation for the other modes are relatively unperturbed. Because no significant changes in THF vibrational modes occur, this suggests that THF begins to solvate the gold surface as well as aspirin, thereby likely weakening the affinity of the aspirin–THF complex to gold without displacing it. The lack of signal intensity changes for the ν_s_C=C feature further indicates that aspirin–THF complexes remain very close to the metal surface and are confined to the strong electric field nearest to the metal surface. Next, the asymmetry of this same vibrational band changes thereby suggesting that a distribution of aspirin–THF clusters is present and that these depend on THF concentration without changing its orientation. It should be noted that other vibrational models should be impacted by the changes in aspirin/THF ratio; however, these are not observed, an effect likely arising from the small intensities, small Raman cross sections, and/or weak/indirect gold–functional group interactions. Finally, additional analyses further support that THF begins to further solvate aspirin and the metal interface without displacing the aspirin–THF complex or changing its orientation with respect to the metal interface. This is reasonable given no observed changes in vibrational intensity and the DFT results shown in Figure 5. Here, the energy-minimized geometries of one aspirin with one to four DFT molecules (similar to experimental results) reveal that the orientation of aspirin relative to gold does not change. Other mechanisms such as restricted vibrational motion or orientation changes can be discounted, as these would lead to an increase or decrease in vibrational intensity, an effect that is not observed. As a result, it is likely that the addition of THF causes its surface density to increase through additional interactions with aspirin or gold, thereby weakening the π-orbital overlap between aspirin and gold without displacing the complex.

## Conclusions

The intermolecular interactions between aspirin, THF–water cosolvent, and gold surface were evaluated to determine how THF enables the SERS detection of aspirin. THF was found to form a hydrogen bonded complex with aspirin, which subsequently promoted the observation of aspirin using SERS. DFT calculations and Raman and SERS spectra supported that the aspirin–THF complex stabilized the carboxylic acid group in aspirin and decreased its hydrophobicity. As a result, THF enabled the adsorption of aspirin–THF to gold in a parallel orientation relative to the interface through π-orbital overlap between the aromatic ring and gold. This interaction weakened depending on the relative amounts of aspirin to THF, whereby additional THF further solvated aspirin and/or the gold surface, a result that weakened the surface affinity of the aspirin–THF complex without displacing it from the metal surface. Overall, rationally selecting cosolvent at varying molar fractions to modulate intermolecular interactions can be a powerful approach to facilitate and modulate adsorption of a nonthiolated analyte to a gold surface, in turn enabling its detection via SERS.

## Supporting Information Available

Details of extinction and Raman spectra, TEM images, and DFT calculations (PDF)
