# Supplementary material for: Effects of Cosolvent on the Intermolecular Interactions between an Analyte and a Gold Nanostar Surface Studied Using SERS
Source: J Phys Chem C Nanomater Interfaces. 2024 Oct 2;128(41):17543–51. doi: 10.1021/acs.jpcc.4c04360 (PMC11492375; doi:10.1021/acs.jpcc.4c04360)
Supplement: Supplementary file 1 — jp4c04360_si_001.pdf [file jp4c04360_si_001.pdf]

The Effects of Co-solvent on the Intermolecular Interactions between an Analyte and Gold Nanostar Surface Studied using SERS

Ryan D. Norton[1], Amanda J. Haes[1]†, and Alexei V. Tivanski[1]**

[1]Department of Chemistry, University of Iowa, Iowa City, Iowa 52242, United States

† This work was performed while AJH was working at the University of Iowa

Table of contents

1. Extinction Spectroscopy of Gold Nanostars in THF	S2-S3
2. TEM of Gold Nanostars in THF with and without Aspirin	S4
3. Raman Measurements for THF Calibration Curve	S5
4. Raman Measurements for Aspirin Calibration Curve	S6
5. Raman Measurements for Three-Component Mixture of Aspirin, THF and Water	S7
6. Equilibrium Geometry of Aspirin's Solvation Shell by DFT	S8
7. Extinction Spectroscopy of Gold Nanostars in THF with Aspirin	S9
8. References	S10

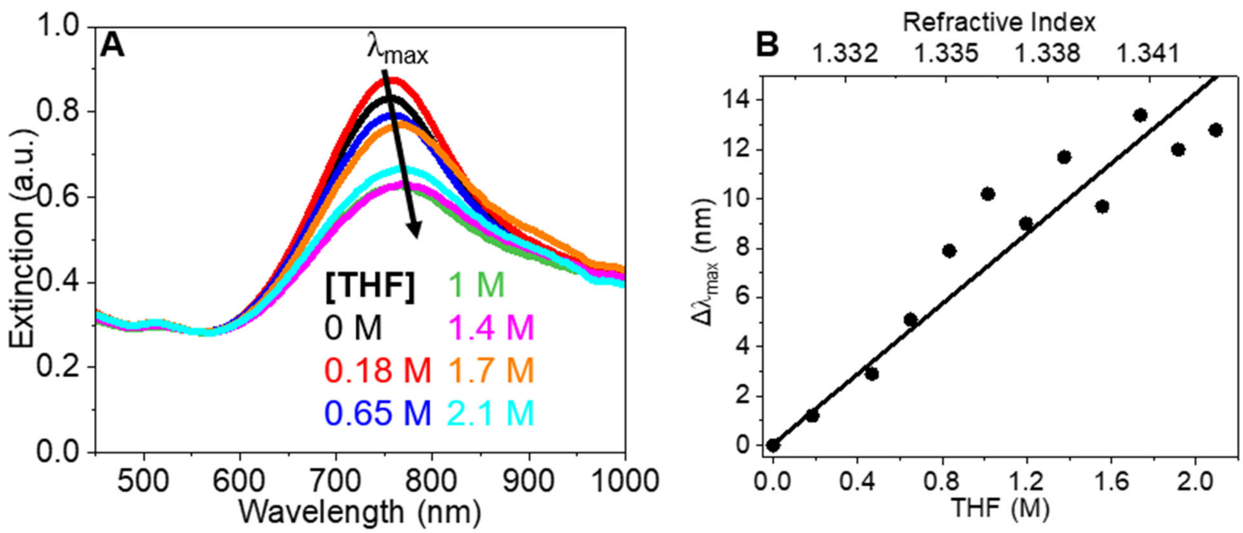

Figure S1. A) Extinction spectra for 0.93 nM gold nanostars at pH 7 after the addition of THF to water with the concentration range of 0- 2.1 M. B) A plot of $\Delta\lambda_{max}$ as a function of THF concentration and corresponding solution refractive index correlated to a linear fit (solid black line) with R^2= 0.904.

Figure S1 shows extinction spectra that exhibit a linear red-shift in λ_{max} from 754.7 to 766.8 nm and a non-linear increase in FWHM from 182 to 240 nm as THF mole fraction increases from 0 to 0.043. The red-shift of the branch-like plasmon can typically be attributed to lengthening of the branches,[1] or a non-morphological change, like chemical changes on the nanoparticle surface. A 25% decrease in extinction is observed as THF mole fraction increases from 0-0.043 throughout the study indicating the loss of nanostars within solution. This drop in extinction is paired with physical observations of solution color loss and particles on the bottom of the vial to claim that sedimentation is the primary consequence of increasing THF concentration. Physical observation of the solutions an hour after measurement concluded revealed a clear solution with all stars settled to the bottom.

Extinction spectra are typically used to understand the nanostar LSPR and aggregation state through key parameters like FWHM of and flocculation area near the hybridized branch plasmon.[2] The FWHM broadens by 30% above 0.015 mole fraction THF and the flocculation area remains constant throughout the range of 0-0.045 mole fraction THF. The increase in FWHM could be attributed to the growth of a new cluster plasmon, but the flocculation area would be expected to increase if this was supported.[2] As the flocculation area remains constant, we can conclude that there is no significant cluster formation, likely no significant change in nanostar stability, throughout our experimental timeframe. The linear change in λ_{max} is attributed to the change in local refractive index as THF concentration increases. Directly changing the refractive index near the surface as bulk concentration of THF increases indicates that THF is present near the surface, but not necessarily bound to the surface.

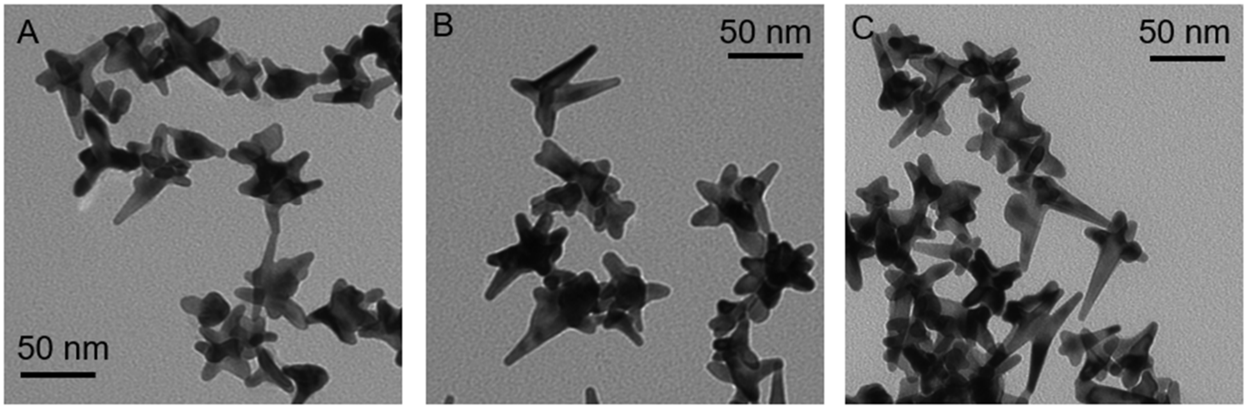

Figure S2. TEM images of activated, EPPS-stabilized, gold nanostars exposed to A) water, B) 2 M THF, and C) 2 M THF with 68 mM aspirin for 12 minutes before dilution by half in ethanol and dried on copper TEM grids coated with Formvar and carbon.

Figure S2 shows TEM images representative of the entire sample for stars exposed to water, 2 M THF, and 68 mM aspirin in 2 M THF where the branch length and radius of curvature were analyzed for each sample. In this case, the morphology of the nanostars does not change as the branch length and radius of curvature show no statistically significant change from 20.8 ± 4.2 nm and 3.4 ± 0.2 nm to 21.3 ± 5.3 nm and 3.3 ± 0.2 nm indicating that the red-shift observed with extinction spectroscopy in Figure S1 does not indicate restructuring. TEM analysis shows no significant difference between gold nanostars in 0 and 0.043 mole fraction THF, determined through one-way analysis of variance.

TEM images for gold nanostars in 0-0.043 mole fraction THF with 68 mM aspirin show a branch length varying from 20.8 ± 4.2 to 21.9 ± 4.8 nm and a radius of curvature from 3.4 ± 0.2 nm to 3.5 ± 0.3 nm. Both are found to not be statistically different utilizing one-way analysis of variance.

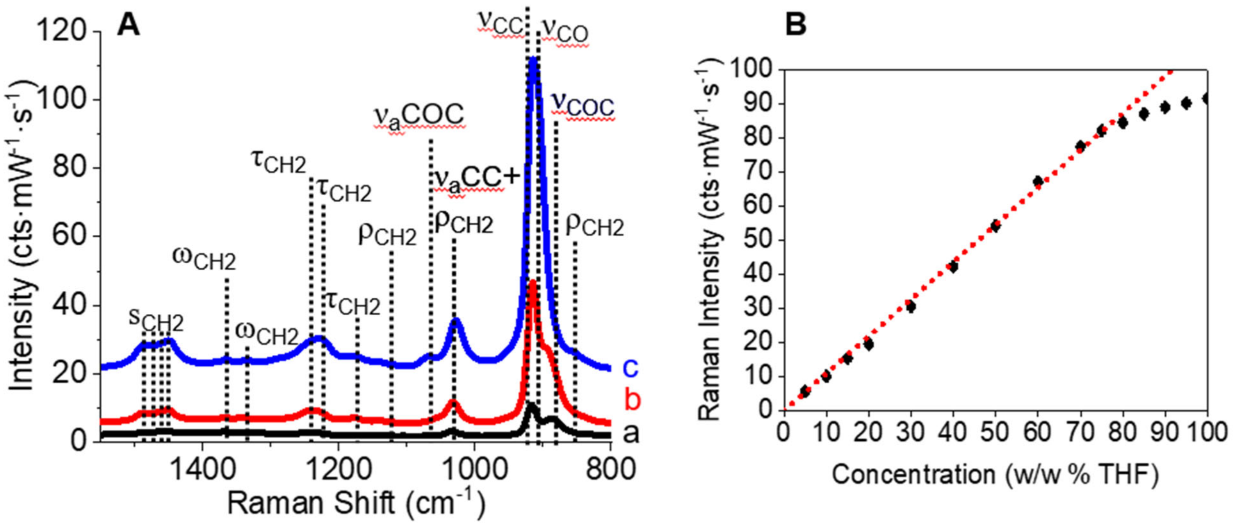

Figure S3. A) Raman spectra of a) 1.4 M THF in water, b) 5.3 M THF in water, and c) neat (12.3 M) THF.[3] B) Raman intensity of the THF band envelope near 914 cm^{-1} as a function of THF concentration. The linearity of the calibration curve (dotted red line, $R^2 = 0.996$) is lost near 75% THF where the azeotrope is ~78% THF. The plots of the individual modes within the band envelope shows that the ν_{CC} and ν_{CO} show similar slopes, but the ν_{COC} shows a drastically lower slope by concentration of THF. Spectra collected at 35 seconds, 10 averages, and 33.5 mW with a 785 nm laser Spectra were offset for clarity (+5 for spectrum b and +22 for spectrum c).

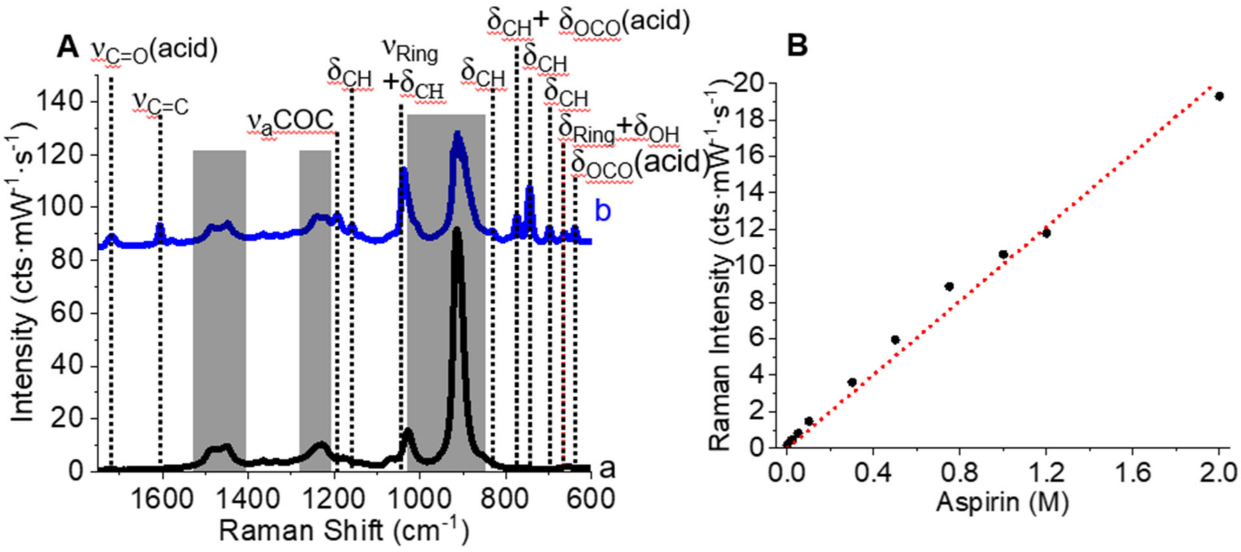

Figure S4. A) Normal Raman spectra of a) neat THF and b) 2 M aspirin in neat THF. Gray stripes indicate THF unique vibrational modes while all aspirin vibrational modes are explicitly noted.[4, 5] Spectra collected at 35 seconds, 10 averages, and 33.5 mW with a 785 nm laser. Spectra were offset for clarity (+85 for spectrum b). B) Calibration curve for Raman intensity of the most intense, unique aspirin mode of δ_{CH} at 753 cm^{-1} plotted as a function of aspirin concentration, along with the linear fit (dotted red line, $R^2 = 0.994$).

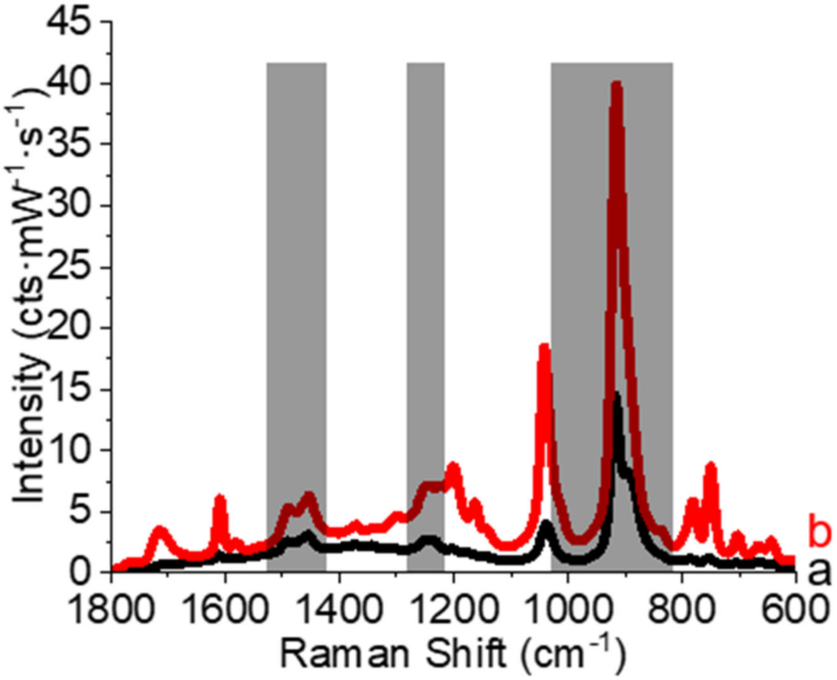

Figure S5. A) Normal Raman spectra of a) the top phase after liquid-liquid phase separation and b) the bottom phase after LLPS. All features highlighted in gray are attributed to THF while non-highlighted features are attributed to aspirin (band assignments and frequencies displayed in Figures S3 and S4). Spectra collected at 30 seconds, 10 averages, and 32.7 mW with a 785 nm laser.

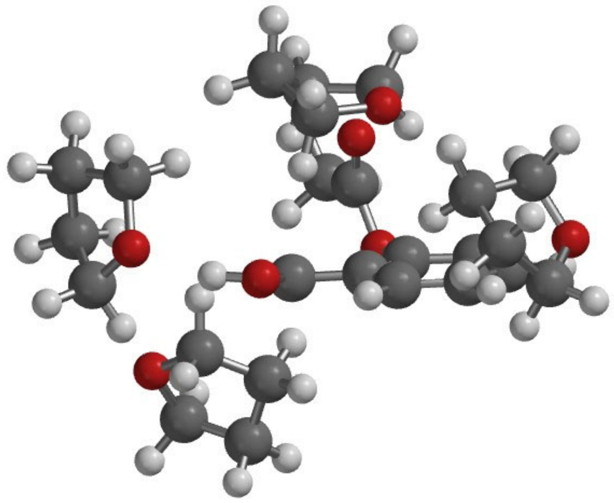

Figure S6. DFT calculations depicting equilibrium geometry the solvation shell of aspirin at high concentration in THF and water. The ratio of molecules is one aspirin: four THF: twenty water molecules or 1.5 M aspirin in 5.8 M THF. Water molecules have been removed from the image to clarify the interactions between aspirin and THF.

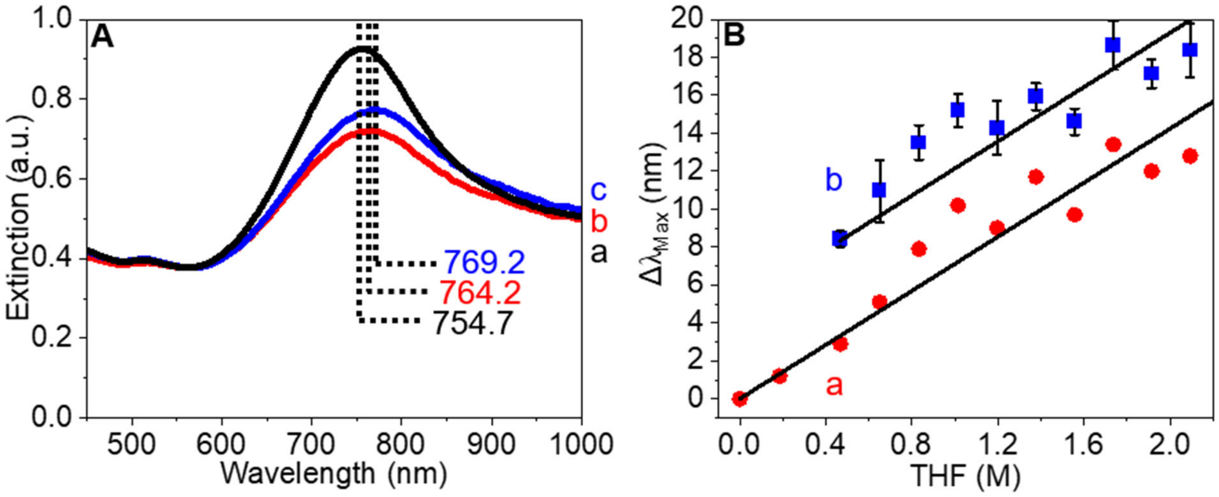

Figure S7. A) Extinction spectra for 0.93 nM gold nanostars a) in water, b) after the addition of THF to final concentration of 1 M THF, and c) after the addition of aspirin and THF to final concentration of 68 mM aspirin and 1 M THF. Vertical dotted lines and numbers denote the λ_{max} of each extinction spectrum. The color of the reported λ_{max} correlates to the color of each spectrum the value is extracted from. B) A plot of $\Delta\lambda_{max}$ of a) 0.93 nM gold nanostars in THF/water and b) 0.93 nM gold nanostars with 68 mM aspirin in THF/water plotted as a function of THF concentration referenced to of 0.93 nM gold nanostars in water. Black lines represent linear fits for each set of data with R^2 = 0.973 (red) and 0.989 (blue).

References

(1) Harder, R. A.; Wijenayaka, L. A.; Phan, H. T.; Haes, A. J. Tuning gold nanostar morphology for the SERS detection of uranyl. *Journal of Raman Spectroscopy* **2021**, *52* (2), 497-505.
(2) Phan, H. T.; Heiderscheit, T. S.; Haes, A. J. Understanding Time-Dependent Surface-Enhanced Raman Scattering from Gold Nanosphere Aggregates Using Collision Theory. *J Phys Chem C Nanomater Interfaces* **2020**, *124* (26), 14287-14296.
(3) Cadioli, B.; Gallinella, E.; Coulombeau, C.; Jobic, H.; Berthier, G. Geometric structure and vibrational spectrum of tetrahydrofuran. *The Journal of Physical Chemistry* **1993**, *97* (30), 7844-7856.
(4) Boczar, M.; Wójcik, M. J.; Szczeponek, K.; Jamróz, D.; Zięba, A.; Kawałek, B. Theoretical modeling of infrared spectra of aspirin and its deuterated derivative. *Chemical Physics* **2003**, *286* (1), 63-79.
(5) C. Muthuselvi, M. D. a. S. P. Growth and Characterization of Aspirin Crystal in the Phosphoric acid Medium. *Journal of Chemical and Pharmaceutical Research* **2016**, *8* (5), 804-814.
